# The serpentine mitral valve and cerebral embolism

**DOI:** 10.1186/1476-7120-9-7

**Published:** 2011-02-27

**Authors:** James Ker

**Affiliations:** 1 Department of Physiology, University of Pretoria, Pretoria, South Africa, PO Box 24318, Gesina, Pretoria, 0031 South Africa

## Abstract

Valvular strands, well-delineated filiform masses, attached to cardiac valve edges are associated with cerebral embolism and stroke. Strokes, caused by emboli from valvular strands, tend to occur among younger persons.

In this case report a valvular strand, giving a peculiar serpentine appearance to the mitral valve is described. This mitral valvular strand was the only explanation for an episode of cerebral embolism, presenting with a transient right sided hemiparesis.

It is proposed that a randomized study involving combined treatment with aspirin and clopidogrel is warranted in young patients with valvular strands, presenting with a first episode of cerebral embolism.

## Introduction

Valvular strands have been described as small, well-delineated masses with a predilection for the valvular endocardium [1]. Clinically these strands present as filiform material attached to cardiac valve edges and is detected by transesophageal echocardiography [2].

These strands, as visualized by transesophageal echocardiography are associated with systemic embolization, especially stroke and notably these strokes tend to occur among younger persons [3,4].

## Case report

A 32 year old man presented with an acute onset of right sided hemiparesis. This occurred within the matter of minutes without any preceding warning symptoms. He had no known illnesses or allergies. He was a non smoker who never had any previous surgery and did not use illicit drugs. He works in the pharmaceutical industry and never experienced any similar symptoms before.

The right sided hemiparesis resolved spontaneously over the next three hours and at the time of clinical examination no objective neurological signs were present. An MRI and MRA scan of the brain and cerebral vasculature were normal. His electrocardiogram and biochemical analysis, including electrolytes, glucose, thyroid function and full blood count were within normal limits. Carotid-IMT and Doppler studies of both carotid arteries were normal. Holter electrocardiography excluded the occurrence of intermittent arrhythmias as a possible cause for embolism. Paradoxical embolism was excluded by the absence of both a patent foramen ovale and deep venous thrombosis. Infective endocarditis was excluded by the absence of positive blood cultures and vegetations.

Transthoracic, two-dimensional echocardiography revealed a peculiar serpentine strand attached to the coapting edge of the mitral valve (see additional files 1, 2 and 3).

He was diagnosed with a valvular strand attached to the mitral valve as the cause for a cerebral embolism to the left mid-cerebral artery. He was treated with a combination of aspirin (100 mg daily) and clopidogrel (75 mg every second day). This maintained his platelet ADP function below 50%. Follow up during the following three years was without any further incidents.

## Discussion

Vilem Dusan Lambl, a Bohemian physician (1824-1895) were the first to describe the occurrence of small, filiform processes he observed on the aortic valve in 1856[5]. Today, these Lambl's excrescences are also referred to as valvular strands and have been observed on all native and prosthetic valves[5]. These strands may occur as single strands, in rows or even in clusters[5]. They can vary in length from 1 mm to 10 mm and are usually less than 1 mm in thickness[5].

Valvular strands are composed of a fibroelastic, avascular core, covered by a layer of endothelial cells[5,6].

The exact pathogenesis of formation of these structures are still unclear, however current opinion is that the initiating factor is that of an endocardial lesion in areas of trauma and/or high shear stress[5,6]. These denuded areas are then covered by fibrin with subsequent covering by an endothelial layer[5,6]. The prevalence of valvular strands has been estimated as 5.5% in a general population referred for transesophageal echocardiography and 40% in patients with stroke of unknown cause[1,2].

The differential diagnosis for valvular strands includes the following[5]: a myxoma, thrombi, valvular vegetations, nonbacterial thrombotic (marantic) endocarditis, cardiac metastases, a fibroelastoma and other primary cardiac neoplasms.

Of all of the above, the most difficult distinction is that between a valvular strand and a fibroelastoma[5,7]. Histologically, these two entities are very similar with both containing a central core of elastic connective tissue, covered by endothelium. However, valvular strands are covered by a single layer of endothelial cells, but fibroelastomas contain regions of multiple layers of endothelial cells[5,7].

Echocardiographically, fibroelastomas are more bulky, with stalks or pedestals sometimes present and multiple, fingerlike projections on their surface[5]. As fibroelastomas are usually found on the mechanically less strained parts of valves and endocardium they tend to be larger than valvular strands[5]. Valvular strands (Lambl's excrescences) are always found on the affected valve's line of closure and this limits their growth[5].

Several published case reports have shown that valvular strands are associated with emboli to the coronary, pulmonary, spinal, retinal and cerebral circulation[1].

Specifically regarding stroke, numerous reports have demonstrated an association with valvular strands, particularly in young patients[3,4,8,9]. The mechanism for embolic events is either that of thrombi forming on the strands which then embolize or it is possible that the valvular strand itself can embolize[2]. Direct visualization of thrombus on a valvular strand have indeed been described before[10].

In conclusion, a case of a valvular strand, attached to the coapting edge of the mitral valve is presented, giving a serpentine appearance to the mitral valve. This valvular strand was the cause for a cerebral embolism which presented with a transient right sided hemiparesis. This is the only current case in the literature, where the combination of aspirin and clopidogrel is used for the prevention of further episodes of cerebral embolism. In the only randomized treatment study to date, no difference in relation to efficacy of warfarin compared to aspirin was found in patients with valvular strands and previous embolic episodes[2]. For this reason a combination of antiplatelet therapy was initiated as a therapeutic trial.

It is proposed that a randomized controlled study involving the combination of aspirin and clopidogrel is warranted in patients with valvular strands presenting with a first episode of cerebral embolism.

## Competing interests

The author declares that they have no competing interests.

## Supplementary Material

Additional file 1 **Serpentine mitral valve**. Transthoracic echocardiographic image. Note the mitral valvular strand, marked with +. Click here for file

Additional file 2 **Serpentine mitral valve**. This is another transthoracic echocardiographic image of the same mitral valvular strand, marked with +. Note the difference in endoventricular position, compared with additional file 1, clearly demonstrating the mobile nature of the strand. Click here for file

Additional file 3 **Serpentine mitral valve**. This is a movie clip, demonstrating the mobile nature of the mitral valvular strand, giving a peculiar serpentine appearance to the mitral valve. Click here for file
