# Supplementary figures and images for: The serpentine mitral valve and cerebral embolism
Source: Cardiovasc Ultrasound. 2011 Feb 27;9:7. doi: 10.1186/1476-7120-9-7 (PMC3051887; doi:10.1186/1476-7120-9-7)

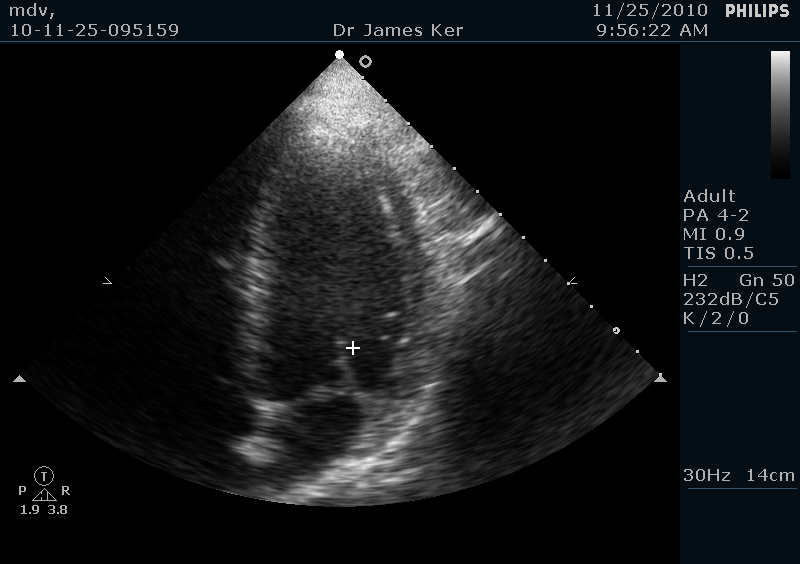

Supplement: Additional file 1 — Serpentine mitral valve. Transthoracic echocardiographic image. Note the mitral valvular strand, marked with +. [file 1476-7120-9-7-S1.BMP]

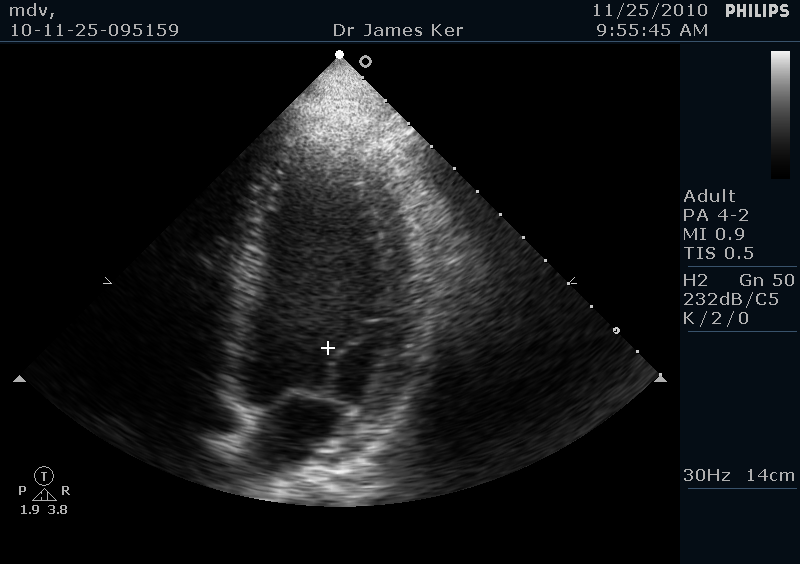

Supplement: Additional file 2 — Serpentine mitral valve. This is another transthoracic echocardiographic image of the same mitral valvular strand, marked with +. Note the difference in endoventricular position, compared with additional file 1, clearly demonstrating the mobile nature of the strand. [file 1476-7120-9-7-S2.BMP]
